# Keynote 1: Moving Social Work: a new evidence-based and co-produced programme to promote physical activity to disabled people

**DOI:** 10.1093/eurpub/ckad133.001

**Published:** 2023-09-11

**Authors:** Brett Smith

**Affiliations:** Department of Sport and Exercise Sciences at Durham University, UK

## Abstract

There is a growing amount of research focused on Health Professionals as valuable messengers and advocates of physical activity. Around the world programmes have now been established to train these professionals in health enhancing physical activity conversations. There are however other professionals that could also be valuable physical activity messengers and advocates. One untapped group is Social Workers. This talk presents Moving Social Work. This is a co-produced 5-year funded project that aims to embed physical activity in social work education, policy, and practice so that these care professionals can work with disabled people to be more physically active. In this keynote address I discuss why social workers are neglected yet important messengers and advocates of physical activity for disabled people. Next, I share insights into how the content of the training was co-produced with disabled people and social workers, what the training involves, and how it is has been implemented. Results of the training are presented. The talk also offers some brief reflections on co-production, disability, and physical activity. It closes with invitations for action that span science, policy, and practice.

